# Conquering the Challenge: Difficult Gallbladders and Delicate Solutions

**DOI:** 10.7759/cureus.106388

**Published:** 2026-04-03

**Authors:** Srija Koripella, Naphene N, Muktesh B, Prakash Dave, Shashirekha CA, Krishna Prasad, Sreeramulu PN, Ravikiran HR

**Affiliations:** 1 General Surgery, Sri Devraj Urs Medical College, Kolar, IND

**Keywords:** bailout procedure, cholelithiasis, difficult laparoscopic cholecystectomy, gallstone cholecystitis, post cholecystectomy bile duct injury, subtotal laparoscopic cholecystectomy

## Abstract

Background

Laparoscopic cholecystectomy is the accepted standard treatment for symptomatic gallstone disease. However, severe inflammation, fibrosis, adhesions, or anatomical distortion can obscure critical landmarks and increase the risk of bile duct injury. In such cases, timely recognition of operative difficulty and appropriate modification of technique are essential to ensure patient safety.

Methods

This prospective case series was conducted at a tertiary care center, R L Jalappa Hospital & Research Center, Tamaka, Karnataka, India, in 2024 and included seven adult patients whose intraoperative findings met predefined criteria for difficult laparoscopic cholecystectomy. Demographic data, operative challenges, technical adaptations, postoperative outcomes, and 30-day follow-up findings were documented and analyzed descriptively.

Results

Operative difficulty was associated with a frozen Calot’s triangle, dense omental or bowel adhesions, a mucocele with impacted stones, a short cystic duct, severe fibrosis, and vascular anomalies. Structured bailout strategies were employed based on intraoperative findings. These included fundus-first (dome-down) dissection, fenestrating subtotal cholecystectomy, selective partial cystic ductotomy for impacted stone retrieval, endoloop ligation of the cystic duct stump, and judicious drain placement. In one patient, transient postoperative jaundice resolved spontaneously without intervention. No bile duct injuries or major postoperative complications occurred. All patients recovered satisfactorily, and no delayed morbidity was identified at the 30-day follow-up.

Conclusion

Difficult laparoscopic cholecystectomy requires vigilance, thorough anatomical knowledge, and the readiness to adapt the operative plan. Familiarity with bailout techniques and individualized intraoperative decision-making can prevent major biliary injuries and enable the safe completion of surgery, even in challenging operative fields.

## Introduction

Laparoscopic cholecystectomy (LC) is widely recognized as the standard surgical treatment for symptomatic gallbladder disease, which may be cholecystitis or cholelithiasis. However, LC can become technically challenging in cases of a “difficult gallbladder,” typically characterized by severe inflammation, fibrosis, dense adhesions, or distorted anatomy. These pathological changes obscure critical landmarks such as Calot’s triangle, thereby increasing the risk of bile duct injury (BDI), one of the most serious complications of LC [1,2].

Difficult LC (DLC) involves a range of intraoperative challenges that can result in prolonged operative duration, higher conversion rates to open surgery, and increased postoperative morbidity. Although no standardized definition exists, DLC is commonly associated with acute or chronic cholecystitis, obesity, previous abdominal surgery, and anatomical variations [1]. Misinterpretation of biliary anatomy remains the leading cause of BDI, underscoring the importance of meticulous dissection and consistently achieving the critical view of safety (CVS). When the CVS cannot be safely obtained due to severe inflammation or distorted anatomy, alternative operative strategies are essential to minimize iatrogenic injury [1,2].

Several bailout techniques have been proposed to enhance safety in such scenarios. Subtotal cholecystectomy, performed either as a fenestrating or reconstituting procedure, is a widely accepted approach that avoids hazardous dissection near the cystic duct and common bile duct. The reconstituting technique has been reported to have lower bile leakage rates compared to fenestrating methods. More recently, the use of laparoscopic linear staplers to close the gallbladder remnant has emerged as a practical and effective strategy, simplifying stump management while potentially reducing postoperative biliary complications [2]. Additional bailout options include fundus-first (dome-down) dissection and conversion to open surgery, with selection guided by intraoperative findings and surgeon expertise [1].

Adjunctive intraoperative imaging modalities, including cholangiography, laparoscopic ultrasonography, and near-infrared fluorescence cholangiography using indocyanine green, have been demonstrated to improve biliary visualization and facilitate safer dissection. However, their routine use may be constrained by limited availability and the requirement for specialized technical expertise. The role of postoperative drainage remains controversial, particularly in acute inflammatory conditions, and should therefore be tailored to the individual patient [1].

Clinical studies have further highlighted the multifactorial nature of DLC. An analysis of 146 patients undergoing DLC demonstrated significant associations between operative difficulty and factors such as acute inflammation, dense adhesions, and anatomical distortion, underscoring the importance of early recognition and appropriate surgical planning [3].

Given the inherent complexity and potential for severe complications in challenging gallbladder cases, familiarity with bailout strategies and adaptive intraoperative decision-making is essential. This case series aims to demonstrate the practical application of these techniques, emphasizing individualized surgical approaches to optimize patient safety and outcomes in DLCs.

## Materials and methods

This was a prospective observational case series conducted at R L Jalappa Hospital & Research Center in Karnataka, India, affiliated to Sri Devraj Urs Medical College in 2024. All procedures were performed under general anesthesia by a single senior surgeon experienced in advanced laparoscopy. The primary objective was to delineate the specific intraoperative challenges and to describe the bailout techniques and technical modifications used to manage them safely.

Study population

The study included adult patients with symptomatic gallstone disease who required cholecystectomy during the study period. Patients were selected for analysis if intraoperative findings met predefined criteria for a “difficult” gallbladder, such as prolonged dissection time, dense adhesions involving the gallbladder or Calot’s triangle, or severe inflammation or fibrosis.

Inclusion Criteria

Adult patients diagnosed with symptomatic cholelithiasis and scheduled to undergo LC were included in the study. Cases were considered eligible when intraoperative findings indicated a DLC, such as the presence of dense adhesions that interfered with safe dissection or a scleroatrophic gallbladder.

Exclusion Criteria

Patients who were considered unsuitable for general anesthesia or had contraindications to laparoscopic surgery were excluded from the study. 

Preoperative evaluation

All patients underwent a standardized preoperative workup, which included a detailed history and physical examination, routine blood tests (complete blood count, liver function tests including alkaline phosphatase and gamma-glutamyl transferase, and serum bilirubin), and abdominal ultrasonography in every case. 

Surgical procedure

A standard four-port LC technique was employed. The initial steps included the establishment of pneumoperitoneum and a routine inspection of the abdomen. Dissection commenced with an attempt to achieve the CVS within the hepatocystic triangle.

Data collection and outcomes

We recorded patient demographics, comorbidities, indications for surgery, and preoperative imaging findings in a structured data collection form. Intraoperative details included the difficulties encountered and the bailout techniques used. Postoperative outcomes assessed included the conversion rate, complications, and length of hospital stay. A 30-day follow-up was conducted to identify any delayed complications.

Ethical considerations

Written informed consent was obtained from all participants prior to enrollment. The study was approved by the Central Ethics Committee of Sri Devraj Urs Academy of Higher Education & Research (reference number: SDUAHER/KLR/R&D/CEC/ S/ PG/ 116 /2024-25). Patient confidentiality was strictly maintained by anonymizing all collected data and restricting access to authorized study personnel.

## Results

Seven patients with DLC were included in this case series. The patients presented with various intraoperative challenges such as anomalous vascular anatomy, dense adhesions, impacted gallstones, frozen Calot’s triangle, and markedly distended gallbladders. Different surgical strategies and bailout techniques were employed based on the intraoperative findings to ensure safe completion of the procedure. A summary of the patient characteristics, intraoperative difficulties, and the corresponding surgical approaches is presented in Table 1.

**Table 1:** Summary of patient characteristics, intraoperative challenges, and surgical strategies used in difficult laparoscopic cholecystectomy.

Case Number	Age, Sex, Diagnosis	Intraoperative Finding/Difficulty	Bailout Strategy Used
Case 1	52 years, male, symptomatic cholelithiasis	Anomalous artery running above cystic duct making Calot’s anatomy unclear	Fundus-first (antegrade/dome-down) dissection
Case 2	35 years, female, acute calculous cholecystitis	Distended gallbladder packed with stones and very short thin cystic duct preventing safe distal clip application	Clip-and-cut technique with endobag and LigaClip
Case 3	65 years, male, cholelithiasis	Thickened inflamed gallbladder with dense adhesions involving omentum and abdominal wall; frozen Calot’s triangle	Fenestrating subtotal cholecystectomy
Case 4	25 years, female, cholelithiasis with mucocele	Markedly distended gallbladder with 3 cm impacted stone at neck and dense adhesions causing frozen Calot’s triangle	Fundus-first approach with partial cystic ductotomy to remove impacted stone
Case 5	88 years, female, cholelithiasis (diabetic)	Massively distended gallbladder with multiple stones and severe fibrosis making clip placement difficult	Fundus-first dissection with endoloop placement on cystic duct stump
Case 6	56 years, female, cholelithiasis	Dense small-bowel adhesions to gallbladder and large stones (2–3 cm)	Gallbladder fenestration with stone extraction, cauterization, and omental patch
Case 7	62 years, female, cholelithiasis (diabetic)	Tense gallbladder with stones and very short cystic duct adherent to cystic artery	Careful dissection achieving Critical View of Safety (standard clipping and division)

Case 1

A 52-year-old man with no comorbidities was found to have an anomalous artery coursing above the cystic duct during laparoscopic cholecystectomy. To safely identify the structures, we performed an antegrade (fundus-first) dissection, starting from the gallbladder fundus and proceeding toward Calot’s triangle. Despite this careful technique, the patient developed transient jaundice on postoperative day 4, which resolved spontaneously. Postoperative magnetic resonance cholangiopancreatography (MRCP) was normal, suggesting only temporary biliary irritation. At the 10-month follow-up, he remained well. This case illustrates the use of a dome-down approach when Calot’s anatomy is unclear.

Case 2

**Figure 1:**
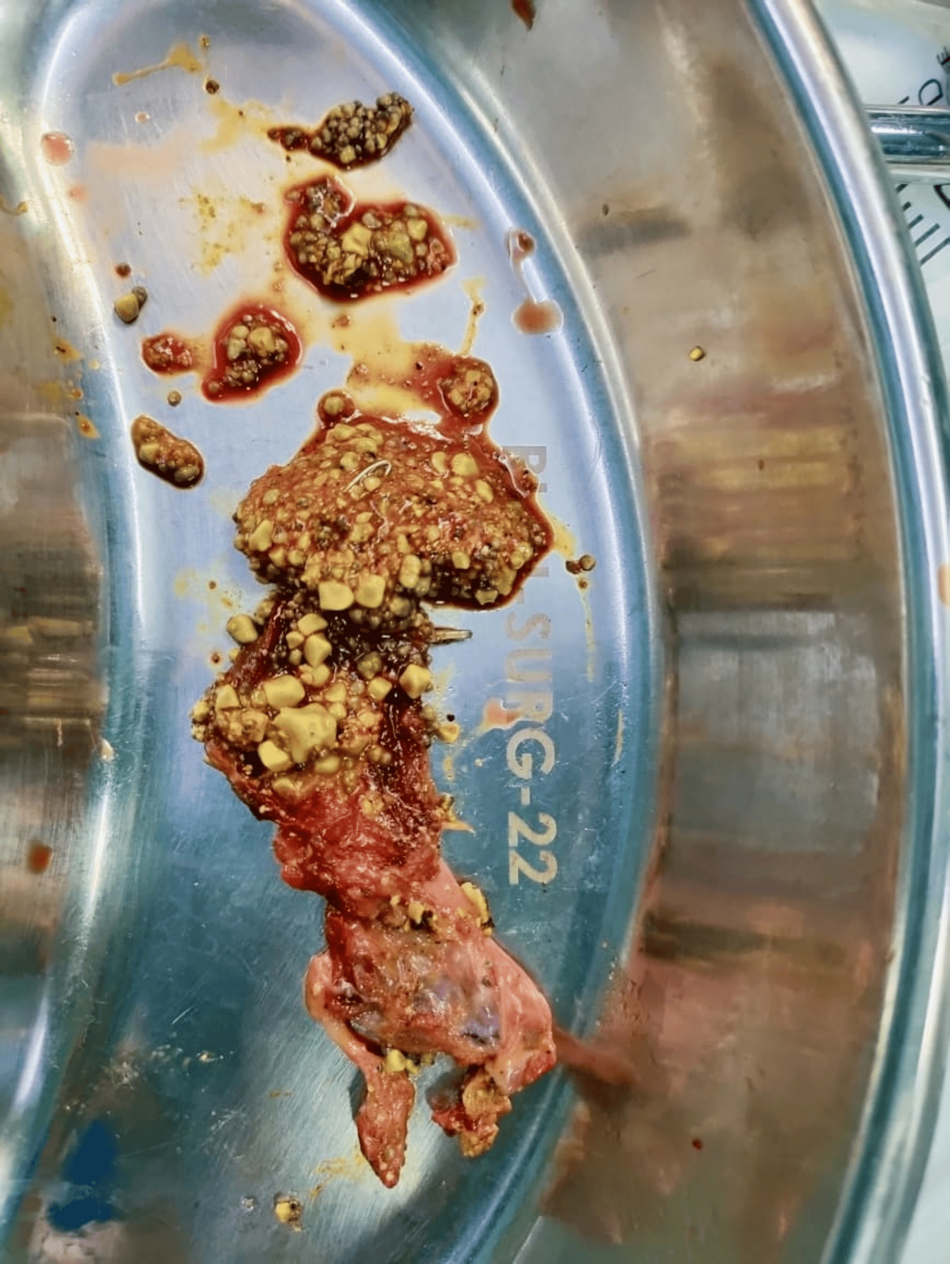
The cut-open gallbladder in Case 2 that was full of stones.

A 35-year-old woman with acute calculous cholecystitis presented with a distended gallbladder packed with stones and a very short, thin cystic duct. The cystic duct was clipped proximally; however, applying a distal clip or loop safely was not possible. We placed the gallbladder in an endobag, then applied a LigaClip (Ethicon, Inc., Raritan, New Jersey, United States) to the stump just above the bag and transected the gallbladder. This “clip-and-cut” technique effectively externalized the gallbladder lumen without bile spillage. Figure 1 shows the extracted gallbladder full of stones. The patient tolerated the procedure well and experienced an uneventful recovery.

Case 3

**Figure 2:**
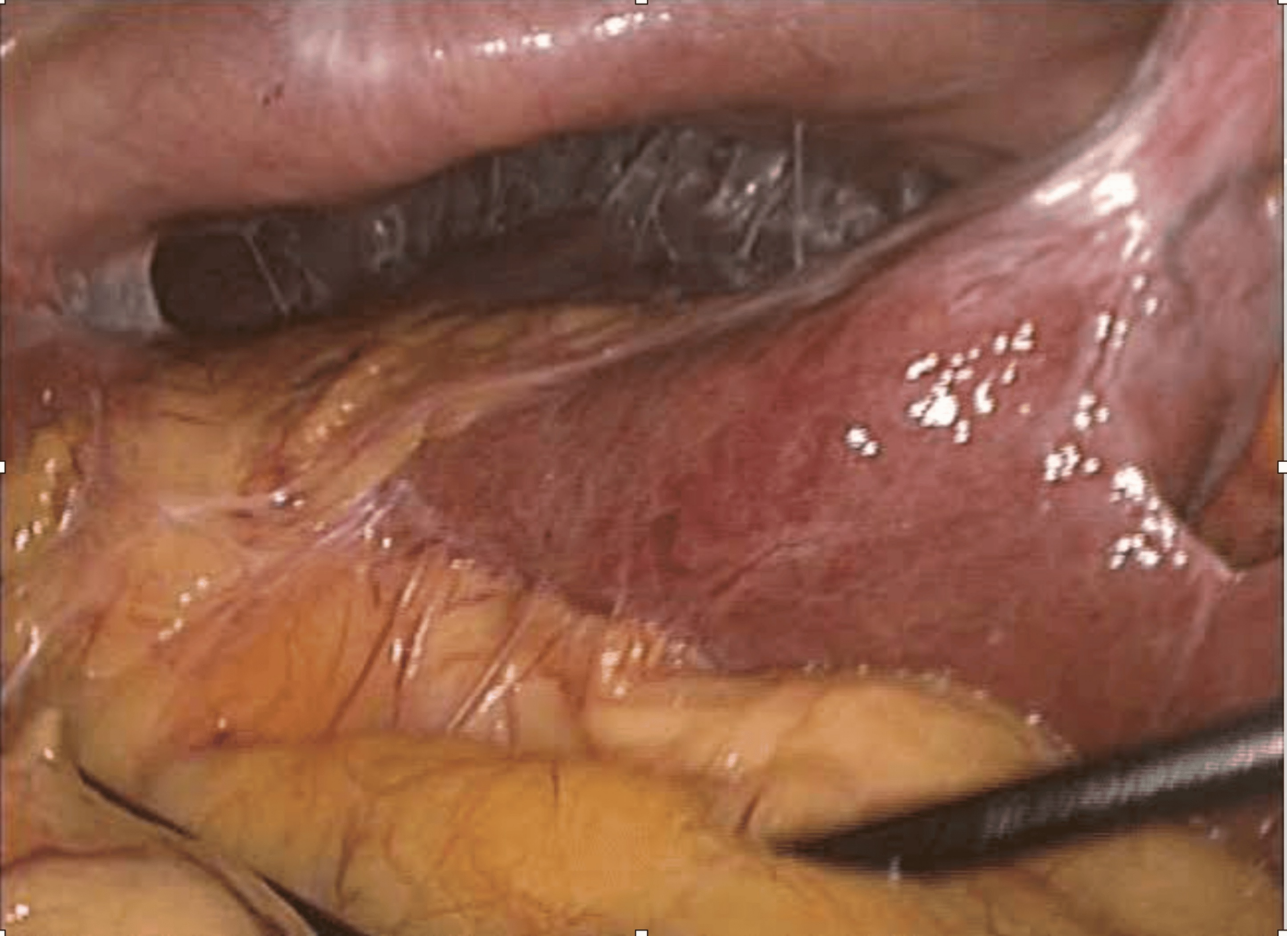
Intraoperative laparoscopic image showing dense adhesions between inflamed gallbladder, omentum, and anterior abdominal wall in Case 3

A 65-year-old man with multiple gallstones was found intraoperatively to have a thickened, inflamed gallbladder with dense adhesions involving the omentum and anterior abdominal wall, obscuring Calot’s triangle as shown in Figure 2. To avoid unsafe dissection, a fenestrating subtotal cholecystectomy was performed. The anterior gallbladder wall was opened, stones were removed, and the posterior wall was left attached to the liver bed. The gallbladder remnant was sutured after mucosal cauterization. The postoperative course was uneventful, and the patient was discharged on postoperative day five. This case demonstrates that subtotal cholecystectomy is an effective bailout strategy in cases of frozen Calot’s triangle anatomy, minimizing the risk of biliary injury.

Case 4

**Figure 3:**
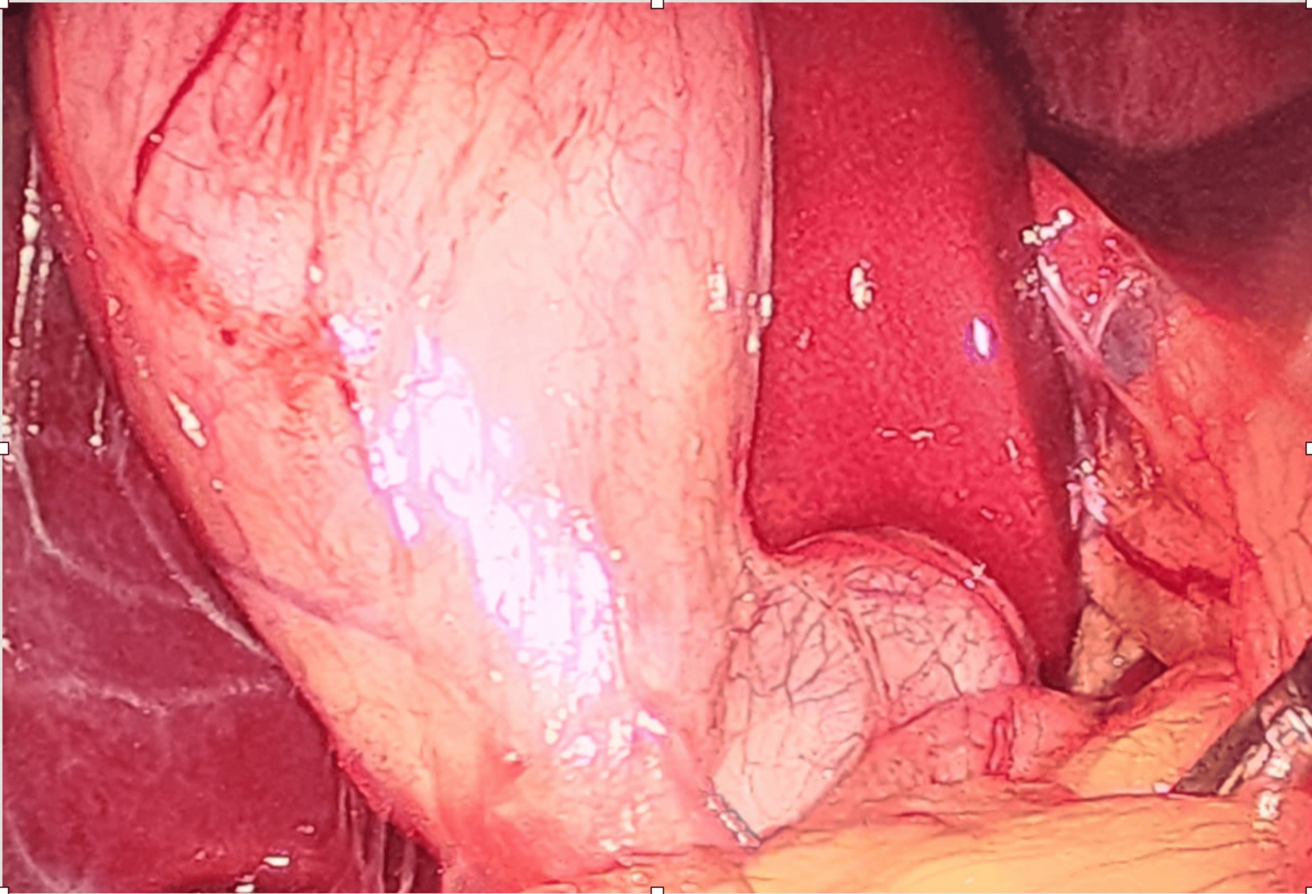
Intraoperative laparoscopic image showing mucocele of gallbladder with impacted stone and frozen Calot's triangle in Case 4.

A 25-year-old female patient presented with intermittent upper abdominal pain and belching for two years. Ultrasonography revealed a 6 mm gallstone. However, intraoperatively, the gallbladder was markedly distended, consistent with a mucocele, and a large 3 cm stone was found impacted at the neck, as seen in Figure 3. Dense and flimsy adhesions surrounded the gallbladder, resulting in a frozen Calot’s triangle.

A fundus-first (antegrade) approach was employed to safely mobilize the gallbladder away from the hepatocystic triangle. The mucocele was decompressed by aspiration; however, the impacted stone prevented adequate grasping and traction. To facilitate extraction, a partial cystic ductotomy was performed: the gallbladder infundibulum was incised just above the duct to retrieve the stone. The cystic duct stump was then securely clipped, and the specimen was removed using an endobag to prevent spillage. The postoperative course was uneventful, with no bile leaks or complications. This case highlights the importance of intraoperative adaptability, demonstrating how a dome-down approach combined with a targeted duct incision can safely manage an impacted stone within a frozen Calot’s triangle.

Case 5

**Figure 4:**
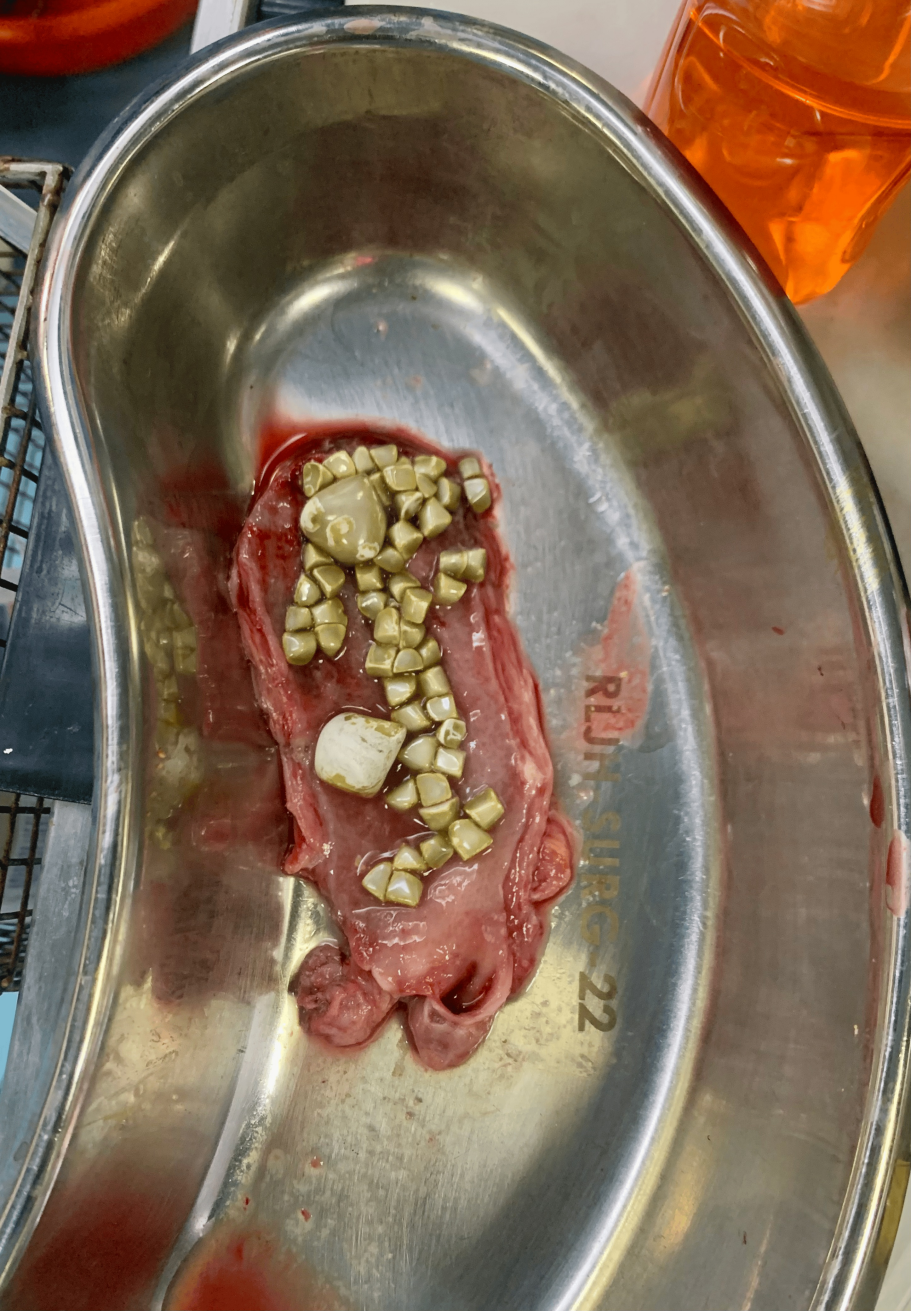
Cut-open gallbladder with stones in Case 5

An 88-year-old woman with diabetes presented with a massively distended gallbladder containing numerous stones and severe fibrosis. We employed a fundus-first dissection technique to carefully free the gallbladder from its bed. Once mobilized, an endoloop was placed around the cystic duct stump to secure it, rather than using clips, before transection. The specimen was then removed without spillage. Figure 4 shows the cut-open gallbladder with stones. The patient recovered well. This case highlights the use of an endoloop for securing the cystic duct remnant when inflammation makes clip application challenging.

Case 6

**Figure 5:**
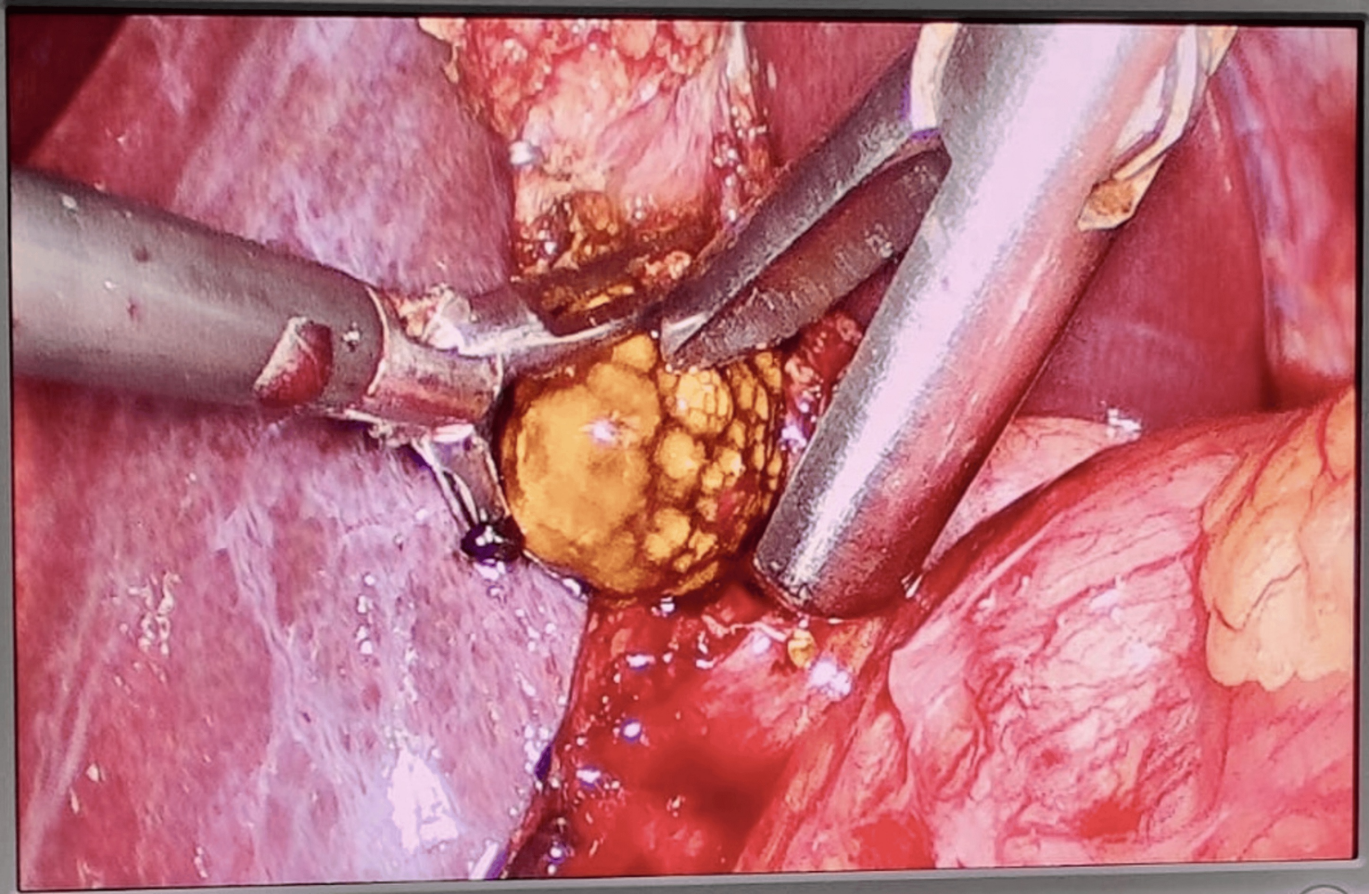
Intraoperative laparoscopic image showing stone extraction using forceps after fenestrating the gallbladder wall in Case 6.

A 56-year-old woman with cholelithiasis presented with dense small-bowel adhesions to the gallbladder and several large stones measuring 2-3 cm. We carefully separated the bowel and laparoscopically fenestrated the gallbladder wall, extracting all stones through this opening, as shown in Figure 5. The remaining gallbladder bed was cauterized and covered with an omental patch (omentum packed over the liver bed). A drain was placed. This technique avoided dissection in the hostile Calot’s triangle. The patient recovered well after surgery.

Case 7

**Figure 6:**
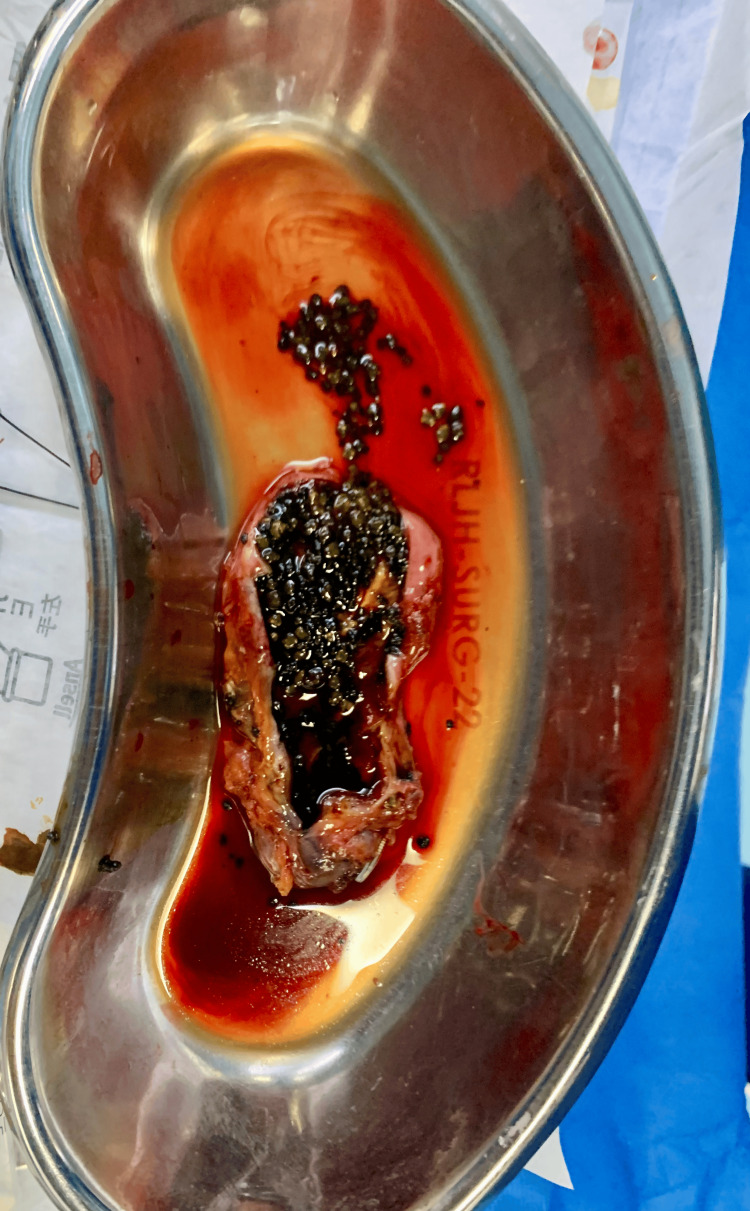
The extracted gallbladder filled with stones and bile in Case 7.

A 62-year-old woman with diabetes presented with a tense, stone-filled gallbladder and a very short cystic duct adherent to its artery. We performed meticulous dissection to achieve the critical view of safety. Once the anatomy was confirmed, the cystic artery and duct were clipped and divided in the standard fashion. No specialized bailout techniques were required. Figure 6 shows the extracted gallbladder filled with stones and bile. The patient had an uneventful recovery. This case demonstrates that careful dissection and obtaining the CVS is possible even in very difficult gallbladders.

## Discussion

LC remains the gold standard for treating symptomatic gallstone disease and is one of the most frequently performed abdominal procedures worldwide. Since its introduction in the late 1980s, LC has largely replaced open cholecystectomy due to its advantages, including reduced postoperative pain, shorter hospital stays, faster recovery, and improved cosmetic outcomes. However, despite these benefits, LC presents unique technical challenges, especially in cases of severe inflammation or distorted biliary anatomy. These situations, commonly referred to as DLC, pose significant concerns for surgeons because of the increased risk of complications, particularly bile duct injury (BDI) [1,4].

BDI remains one of the most serious complications associated with LC, with reported incidence rates ranging from 0.3% to 0.7%, which are higher than those reported during the era of open cholecystectomy [5,6]. Such injuries can lead to significant morbidity, including biliary strictures, cholangitis, and secondary biliary cirrhosis, often necessitating complex hepatobiliary reconstruction. Barrett et al. emphasized that BDI significantly increases postoperative morbidity and healthcare costs, underscoring the need for improved safety strategies during laparoscopic biliary surgery [5]. Misinterpretation of biliary anatomy, rather than technical error, is widely recognized as the most common cause of BDI during LC [4,7]. Consequently, modern surgical practice stresses the importance of structured safety protocols and strict adherence to established anatomical principles.

One of the most important safety concepts in LC is the CVS, first described by Strasberg [7]. The CVS requires three conditions: complete clearance of the hepatocystic triangle of fat and fibrous tissue, separation of the lower third of the gallbladder from the liver bed, and identification of only two structures entering the gallbladder: the cystic duct and the cystic artery. Achieving the CVS significantly reduces the risk of misidentification injuries. However, in difficult cases characterized by severe inflammation, dense adhesions, or fibrosis, achieving the CVS may be technically impossible or unsafe. In such circumstances, surgeons must promptly recognize operative difficulty and adopt alternative strategies to avoid major biliary injury [1,7].

The incidence of DLC varies widely in the literature but has been reported in approximately 10-20% of patients undergoing the procedure [3,8]. Several preoperative and intraoperative factors have been associated with increased operative difficulty. Bat analyzed 146 patients with DLC and reported that dense adhesions, severe inflammation, and distorted anatomy significantly increased operative complexity and operative time [3]. Similarly, Sakuramoto et al. demonstrated that histological evidence of severe inflammatory changes strongly correlates with intraoperative difficulty during LC [9]. Preoperative ultrasonography can also provide predictive information; Daradkeh et al. identified factors such as gallbladder wall thickening, pericholecystic fluid, and impacted stones as indicators of potentially difficult procedures [10].

In this case series, several well-recognized risk factors for DLC were identified, including dense adhesions, distended gallbladders, impacted stones, and anomalous vascular anatomy. These findings align with previously reported predictors of operative difficulty, such as acute cholecystitis, male gender, advanced age, obesity, and prior abdominal surgery [8,11]. Rosen et al. emphasized that these factors are also associated with increased conversion rates from laparoscopic to open surgery [11]. However, advances in surgical techniques and enhanced laparoscopic expertise have reduced conversion rates to approximately 2-6% in experienced centers [12].

One of the key strategies for managing difficult gallbladders is the fundus-first, or dome-down, approach, which was employed in several cases in our series. In this technique, dissection begins at the gallbladder fundus and proceeds toward Calot’s triangle. This approach allows the surgeon to avoid early dissection in the hepatocystic triangle when inflammation obscures anatomical landmarks. Missori et al. described the dome-down technique as a useful alternative in cases of severe adhesions or unclear anatomy, facilitating the safe mobilization of the gallbladder [1]. However, this method requires careful attention to the cystic plate and hepatic parenchyma to prevent bleeding or injury to adjacent structures.

Another important bailout strategy used in our cases was subtotal cholecystectomy, which has gained increasing acceptance as a safe alternative in difficult cases where dissection near the cystic duct is hazardous. Subtotal cholecystectomy can be performed using either a fenestrating or a reconstituting technique. In the fenestrating approach, the anterior wall of the gallbladder is removed while the posterior wall remains attached to the liver bed. The reconstituting technique involves closure of the gallbladder remnant to recreate a small pouch [13]. Studies have demonstrated that subtotal cholecystectomy significantly reduces the risk of BDI compared with attempts at complete cholecystectomy in severely inflamed gallbladders [13,14].

Noda and Kuroki recently described the use of laparoscopic linear staplers to close the gallbladder remnant during subtotal cholecystectomy, reporting favorable outcomes and reduced bile leakage rates in patients with severe cholecystitis [2]. In our series, a fenestrating subtotal cholecystectomy was successfully performed in a patient with a frozen Calot’s triangle, demonstrating its practical value in managing complex anatomy while minimizing operative risk.

Another challenge encountered during DLC is the presence of impacted gallstones in the gallbladder neck or cystic duct, which can significantly restrict traction and visualization. Impacted stones are known to contribute to the so-called “hidden cystic duct syndrome,” where inflammatory adhesions create the appearance of a false infundibulum, potentially leading to misidentification of the common bile duct [5,15]. In such cases, techniques such as gallbladder decompression, partial cystic ductotomy, or stone extraction may help restore anatomical orientation and facilitate safer dissection.

**Figure 7:**
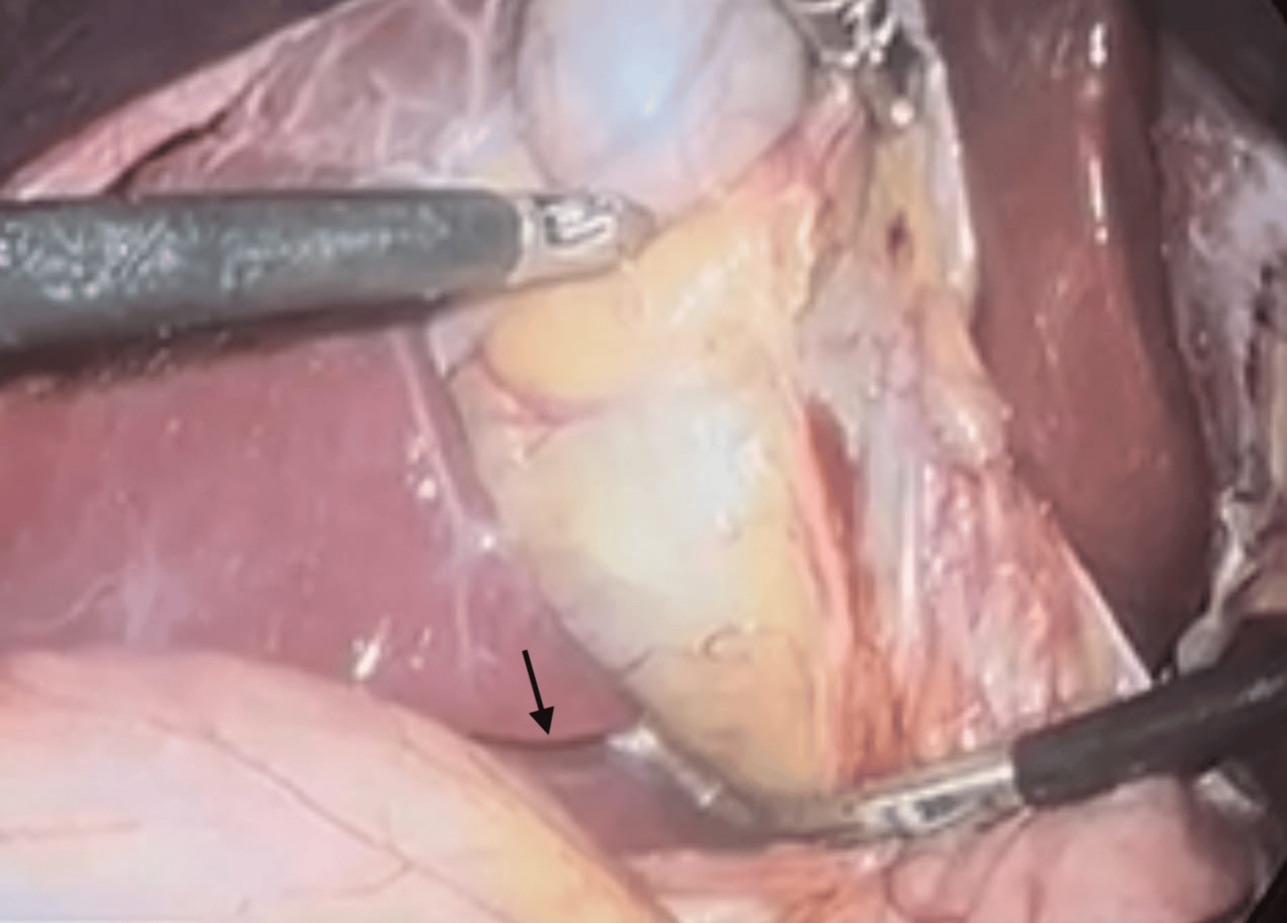
Intraoperative laparoscopic view demonstrating Rouviere’s sulcus (arrow), a key anatomical landmark used to identify the plane of safe dissection during laparoscopic cholecystectomy and to help avoid bile duct injury.

In addition to surgical techniques, anatomical landmarks play a crucial role in preventing biliary injury. Rouvière’s sulcus, as shown in Figure 7, an extrabiliary landmark located on the liver surface, has been identified as a reliable guide for safe dissection during LC.

Studies report that Rouvière’s sulcus is visible in approximately 68-90% of patients and generally indicates the plane above which safe dissection should occur [1,16]. Identifying this landmark, shown in Figure 7, helps surgeons maintain proper orientation and avoid inadvertent injury to the common bile duct.

Technological advances have significantly contributed to enhancing safety during challenging LC procedures. Intraoperative imaging techniques, such as intraoperative cholangiography (IOC), laparoscopic ultrasonography, and near-infrared fluorescence cholangiography using indocyanine green, have been demonstrated to improve visualization of the biliary anatomy [17,18]. Fluorescence cholangiography, in particular, enables real-time identification of biliary structures without requiring duct cannulation and has been increasingly adopted in advanced laparoscopic centers [18]. Although these technologies were not routinely employed in the present study due to resource limitations, their expanding availability may further enhance surgical safety in complex cases.

Another important principle emphasized in the literature is that conversion to open surgery should not be regarded as a failure but rather as a prudent safety measure when laparoscopic dissection becomes unsafe. Berci et al. stressed that the fundamental principle of laparoscopic biliary surgery should always be “first, do no harm,” advocating for early conversion when anatomical uncertainty persists [4]. Delayed conversion after complications arise is associated with worse outcomes compared to timely decision-making during the early stages of operative difficulty [11].

The findings of our case series reinforce the concept that DLC requires not only technical expertise but also sound intraoperative judgment. Structured bailout strategies, such as fundus-first dissection, subtotal cholecystectomy, selective cystic ductotomy, and alternative methods of cystic duct closure, can enable the safe completion of surgery in challenging cases while minimizing the risk of BDI. Importantly, all patients in our series recovered without major complications, and no bile duct injuries were observed, supporting the effectiveness of these safety-oriented approaches.

Limitations

This case series has several limitations that should be acknowledged. The small sample size of seven patients limits the generalizability of the findings and may not represent the full spectrum of clinical scenarios encountered in DLCs. Case series primarily provide descriptive insights rather than statistically robust conclusions; therefore, the results should be interpreted with caution.

Another limitation is that all procedures were performed by a single experienced surgeon at a single tertiary care center. While this approach ensures consistency in operative technique and intraoperative decision-making, it may introduce operator-related bias and limit the generalizability of the results. Surgeons with varying levels of experience or different institutional resources may achieve different outcomes when managing similarly complex cases.

In addition, the observational design of this study and the lack of a comparison group limit the ability to assess the relative effectiveness of different bailout strategies. The choice of surgical technique in each case was guided by intraoperative findings and the surgeon's judgment rather than a standardized protocol. Furthermore, although patients were followed for 30 days postoperatively, longer follow-up may be necessary to detect delayed complications such as retained stones or biliary strictures. Larger multicenter studies with extended follow-up would help strengthen the evidence regarding the optimal management of DLC.

## Conclusions

LC remains the standard treatment for symptomatic gallstone disease; however, the procedure can become technically challenging in the presence of severe inflammation, distorted anatomy, or dense adhesions. The cases presented in this series demonstrate that DLC is relatively common and requires not only technical proficiency but also careful intraoperative decision-making. Notably, in our series, many of these intraoperative difficulties were not anticipated during preoperative assessment and often presented as unexpected challenges during surgery. Major complications, particularly biliary and vascular injuries, are often associated with misinterpretation of anatomy and delayed recognition of operative difficulty. These complications can lead to significant morbidity, underscoring the importance of maintaining a structured and safety-focused surgical approach.

Adherence to fundamental surgical principles, including meticulous dissection, continuous orientation to anatomical landmarks, and achievement of the critical view of safety, remains essential. When safe dissection is not feasible, timely use of bailout strategies such as the fundus-first approach, subtotal cholecystectomy, alternative cystic duct closure methods, or selective drain placement can help prevent major injury. The choice of technique should be guided by intraoperative findings, the degree of inflammation, and the surgeon’s experience. Importantly, conversion to open surgery should be considered a prudent safety measure rather than a procedural failure, ensuring optimal patient outcomes in complex cases.
